# Therapeutic potential of botanical drugs and their metabolites in the treatment of pelvic inflammatory disease

**DOI:** 10.3389/fphar.2025.1545917

**Published:** 2025-04-10

**Authors:** Han-Zhi Zhong, Pei-Jia Yan, Qi-Feng Gao, Jue Wu, Xiao-Li Ji, Shao-Bin Wei

**Affiliations:** ^1^ School of Clinical Medicine, Chengdu University of Traditional Chinese Medicine, Chengdu, China; ^2^ Department of Gynecology, Hospital of Chengdu University of Traditional Chinese Medicine, Chengdu, China

**Keywords:** antimicrobial, *Chlamydia trachomatis*, mechanism, *Neisseria gonorrhoeae*, pelvic inflammatory disease

## Abstract

The application of botanical drugs and their metabolites in the treatment of pelvic inflammatory disease (PID) has garnered significant attention. Owing to their broad-spectrum activity, global accessibility, and structural diversity, botanical drugs have emerged as promising candidates for adjunctive or alternative therapies. This review systematically summarizes botanical drugs and their metabolites, focusing on their antimicrobial potential against endogenous and exogenous pathogens associated with PID. Specifically, it addresses various underlying antibacterial mechanisms, including interference with bacterial cell membranes and cell walls, inhibition of pathogen-specific efflux pumps, modulation of pathogen-related gene expression, and synergistic effects when combined with conventional antibiotics. This review highlights the therapeutic promise of botanical drugs and their metabolites, emphasizing critical findings regarding their inhibitory effects on PID-associated pathogens. Such insights provide valuable guidance for future therapeutic strategies and may support ongoing antibiotic discovery and development.

## 1 Introduction

Pelvic inflammatory disease (PID) is a severe infectious-inflammatory condition affecting the female upper genital tract, characterized by endometritis, salpingitis, oophoritis, and/or pelvic peritonitis (Brunham et al., 2015). It is associated with significant reproductive sequelae, including tubal-factor infertility, ectopic pregnancy, and chronic pelvic pain (Haggerty et al., 2010). The etiology of PID primarily involves polymicrobial infections, notably *Neisseria gonorrhoeae* (Quillin and Seifert, 2018), *Chlamydia trachomatis* (Darville, 2021) and anaerobic or atypical bacteria (Mitchell et al., 2021). Current clinical guidelines advocate broad-spectrum antimicrobial regimens (Smith et al., 2007; Petrina et al., 2019)—typically combining ceftriaxone, doxycycline, metronidazole, and levofloxacin—with a transition to oral therapy after 24–48 h of clinical improvement, typically spanning 14 days (Workowski et al., 2021). While randomized controlled trials and clinical studies demonstrate resolution of symptoms and pathogens with these regimens (Wiesenfeld et al., 2021; Petrina et al., 2019), prolonged antibiotic use raises concerns such as drug hypersensitivity, gut dysbiosis, and emerging multidrug resistance (Petrina et al., 2019; Horton et al., 2015; Scott et al., 2016). Cephalosporins, a cornerstone of PID therapy, are associated with underreported central nervous system-related adverse drug reactions (ADRs) (Cock, 2015; Bhattacharyya et al., 2016; Grill and Maganti, 2011). Surveillance data from the FDA Adverse Event Reporting System (FAERS) reveal a progressive rise in doxycycline-associated ADRs, with 9,641 cases documented between 2003 and 2024 (Xiong et al., 2024). These challenges underscore the urgent need for alternative therapies.

Botanical drugs, particularly those derived from traditional Asian medicine, represent a promising Frontier (Dushime et al., 2020). Natural metabolites from plants such as *Solanum nigrum* [Solanaceae; *S. nigrum* L.] and *Althaea officinalis* [Malvaceae; *A. officinalis* L.] exhibit antibacterial and anti-inflammatory properties historically leveraged in PID management (Khare, 2008). Preclinical studies highlight the efficacy of *Distemonanthus benthamianus* [Fabaceae; *D. benthamianus* Baill] and *Solanum torvum* [Solanaceae; *S. torvum* Sw.] extracts against *N. gonorrhoeae*, with metabolites like esculetin disrupting bacterial cell wall integrity (Obiang et al., 2019). Despite these advances, mechanistic insights into botanical therapies remain fragmented.

This review synthesizes current evidence on phytopharmaceuticals and their metabolites targeting PID-associated pathogens, aiming to identify novel therapeutic strategies while addressing critical research gaps in molecular mechanisms and clinical translatability.

## 2 Scope and terminology

This review involved an extensive search of relevant databases and resources, including PubMed, Embase, China National Knowledge Infrastructure (CNKI), and the Pharmacopoeia of the People’s Republic of China. A combination of Medical Subject Headings (MeSH) and keyword searches was employed, with terms such as “*C. trachomatis*,” “*N. gonorrhoeae*,” “botanical drug,” “pharmacological actions,” “herb,” “herbal medicine,” “pelvic inflammatory disease,” “endometritis,” and “salpingitis.” The search strategy is provided in Supplementary Table 1, with the final search conducted up to 12 November 2024.

Predefined inclusion criteria: 1) Studies focusing on botanical drugs for pelvic inflammatory disease (PID); 2) Studies published in English or Chinese; 3) Phytochemical composition of the herbal intervention was identified. Exclusion criteria: 1) Studies with unavailable full text; 2) Duplicate publications; 3) Non-original research (e.g., reviews, commentaries).

The accuracy of all plant names was confirmed using Plants of the World Online (http://www.plantsoftheworldonline.org). The botanical drug extracts discussed in this study are produced through well-defined extraction and preparation methods. The primary bioactive compounds of these botanical drugs are their metabolites. Furthermore, compound formulations of botanical drugs have been subjected to mass spectrometric analysis, enabling clear identification of their metabolites. All the composition of botanical drugs associating with PID are in Table 1.

**TABLE 1:** The metabolites of botanical drugs associating with PID.

Composition		Medicinal part
Scientific name	Species name	
*Solanum nigrum* L.	*Solanum nigrum*	Dried roots, leaves, and fruits
*Althaea officinalis* L.	*Althaea officinalis*	Dried roots
*Distemonanthus benthamianus* Baill.	*Distemonanthus benthamianus*	Dried roots and bark
*Solanum torvum* Sw.	*Solanum torvum*	Dried roots, leaves, and fruits
*Andrographis paniculata* (Burm.f.) Wall. ex Nees	*Andrographis paniculata*	Dried aerial parts
*Panax ginseng* C.A.Mey.	*Panax ginseng*	Dried roots
*Scutellaria baicalensis* Georgi	*Scutellaria baicalensis*	Dried roots
*Coscinium fenestratum* (Gaertn.) Colebr	*Coscinium fenestratum*	Rhizome
*Helichrysum caespititium* (DC.) Sond	*Helichrysum caespititium*	The whole herb, particularly the flowers and stems
*Ocimum tenuiflorum* L.	*Ocimum tenuiflorum*	The whole herb, particularly the leaves and roots
*Magnolia officinalis* Rehder and E.H.Wilson	*Magnolia officinalis*	Dried bark
*Rheum palmatum* L	*Rheum palmatum*	Rhizome
*Phellodendron amurense* Rupr	*Phellodendron amurense*	Dried bark
*Persea americana* Mill	*Persea americana*	Fruits, leaves, and seeds
*Glycyrrhiza glabra* L	*Glycyrrhiza glabra*	Dried roots and rhizome
*Trachyspermum ammi* (L.) Sprague	*Trachyspermum ammi*	Seeds
*Centella asiatica* (L.) Urb	*Centella asiatica*	The whole herb, particularly the leaves and roots
*Zanthoxylum nitidum* (Roxb.) DC	*Zanthoxylum nitidum*	Fruits and bark
*Panax ginseng* C.A.Mey	*Panax ginseng*	Dried roots
*Persea lingue* (Ruiz & Pav.) Nees	*Persea lingue*	Dried bark
*Fridericia platyphylla* (Cham.) L.G.Lohmann	*Fridericia platyphylla*	Dried stems and leaves
*Vernonanthura serratuloides* (Kunth) H.Rob	*Vernonanthura serratuloides*	Dried herb
*Campsis grandiflora* (Thunb.) K. Schum	*Campsis grandiflora*	Dried flowers, stems and leaves
*Mallotus philippensis* (Lam.) Müll.Arg	*Mallotus philippensis*	Fruit glandular trichomes and stems
*Indigofera suffruticosa* Mill.	Fabaceae	Dried leaves
*Chimonanthus salicifolius* S.Y.Hu	Calycanthaceae	Dried leaves
*Monoon longifolium* (Sonn.) B.Xue and R.M.K.Saunders	Annonaceae	Dried leaves, bark or stems

PS: The “Scientific name” and “Species name” in the table were retrieved from the “Medicinal Plant Names Services” website (https://mpns.science.kew.org/mpns-portal); “Name in pharmacopeia” was retrieved from National Pharmacopoeia Committee.

## 3 Pathogens of PID

The common pathogens of PID can be briefly categorized into two groups based on the route of causation: 1) exogenous pathogens, primarily sexually transmitted microorganisms such as *N*. *Gonorrhoeae*, *C*. *trachomatis* and *Mycoplasma genitalium*; 2) endogenous pathogens, which include bacteria associated with bacterial vaginosis (BV) as well as other anaerobic, parthenogenetic, and aerobic microorganisms, such as *Haemophilus influenzae*, *Escherichia coli* and *Mycobacterium anisopliae*. The co-occurrence of microorganisms from both categories can lead to synergistic interactions that exacerbate the clinical progression of the disease. For instance, the presence of aerobic bacteria can cause tissue necrosis and induce anaerobic conditions, which, in turn, promote the growth of anaerobic bacteria and the formation of tubo-ovarian abscesses (Sweet, 1975).

### 3.1 Exogenous pathogens

*C*. *trachomatis* is the most prevalent cause of bacterial sexually transmitted infections worldwide, accounts for an estimated 131 million new cases each year (Newman et al., 2015). As an obligate intracellular bacterium, *C. trachomatis* has a specialized life cycle that predisposes it to persistent or recurrent infections (Brunham and Rey-ladino, 2005; Paavonen, 2012). A significant portion of these infections, particularly among women, are asymptomatic or subclinical, with silent carriers constituting up to 70% of the female cases (Hoenderboom et al., 2017). When untreated, *C*. *trachomatis* can ascend from the lower genital tract to affect the uterus, fallopian tubes, and ovaries, frequently leading to PID (Haggerty et al., 2010). Such developments considerably increase the risk of severe reproductive health issues, including ectopic pregnancies and infertility. The link between *C*. *trachomatis* infections and these detrimental reproductive health outcomes has been further validated by recent research (Oakeshott et al., 2010; Davies et al., 2016; Reekie et al., 2018).

*N*. *Gonorrhoeae,* a Gram-negative diplococcus bacterium, predominantly targets the genitourinary system, as well as the rectum and pharynx. Many of these infections are asymptomatic, complicating detection and treatment efforts (Keshvani et al., 2019). Without appropriate therapeutic intervention, the bacterium can lead to severe health issues including tubal infections, PID, ectopic pregnancies, infertility, and even cause blindness in neonates. Notably, *N*. *Gonorrhoeae* stands as the second most widespread bacterial sexually transmitted infection worldwide, trailing only *C*. *trachomatis* (Unemo et al., 2019)*,* with an alarming rise in cases from 78 million in 2012 to 87 million by 2016 (Rowley et al., 2019; Newman et al., 2015). The emergence of strains resistant to critical antibiotics such as ceftriaxone and azithromycin has been documented, signaling a pressing challenge in the treatment landscape (Jennison et al., 2019; Eyre et al., 2019). This situation has heightened the urgency for innovative public health strategies and the development of alternative therapeutic measures, particularly in light of limited effective antibiotic options (Tedijanto et al., 2020). Accordingly, the World Health Organization (WHO) has classified *N*. *Gonorrhoeae* as a “priority pathogen,” underscoring the critical need for new therapeutic avenues to manage this persistent public health threat (Tacconelli et al., 2018).

### 3.2 Endogenous pathogens

Bacteria associated with bacterial vaginosis is characterized by a transformation of the vaginal microbiota, shifting from a predominance of *Lactobacillus* species to a varied composition featuring parthenogenetic and anaerobic microorganisms (Hillier et al., 1996). This alteration is associated with the pathogenesis of PID. Specifically, anaerobic Gram-negative bacilli, frequently identified among the BV-associated microbiota, have been isolated from the upper genital tract in patients suffering from endometritis and salpingitis, highlighting their potential contribution to the development of PID. In a study encompassing 545 women with symptoms indicative of PID, significant associations were found between the detection of BV-related genera and species like *Sneathia*, *Atopobium vaginae* and BVAB1 in cervical or endometrial samples, and the histological confirmation of endometritis, using PCR techniques (Haggerty et al., 2016). In a similar vein, research conducted in Kenya noted that individuals diagnosed with salpingitis were more likely to exhibit PCR-detected BV-associated bacteria in their tubal samples than control subjects without salpingitis (Hebb et al., 2004). Moreover, a comprehensive longitudinal study involving 2,958 participants demonstrated that a diagnosis of BV, based on either Amsel criteria or Nugent score, was linked with an elevated risk of developing PID (Turpin et al., 2021). Another longitudinal cohort study underscored that the presence of BV-associated microorganisms, as determined through culturing techniques, doubled the incidence of PID among the studied population (Ness et al., 2005).

In many instances, PID has been identified as polymicrobial in nature. In Kenyan patients with laparoscopically confirmed acute salpingitis, analysis of tubal specimens revealed the presence of 3–10 distinct phylotypes of 16S rRNA DNA, including organisms typically associated with the oropharyngeal and gastrointestinal tracts, in addition to those linked to BV (Hebb et al., 2004). Investigations into participants with laparoscopically confirmed tubal infections have detected a variety of anaerobic organisms (including *Mycobacterium* spp., *Clostridium* spp.) and parthenogenetic or aerobic organisms (such as *E*. *coli*, *Streptococcus* spp., *Staphylococcus spp.*, *H. influenzae*) isolated via culture methods from the fallopian tubes or peritoneal samples.

## 4 Antibacterial effect of botanical drugs and its metabolites on exogenous pathogens

### 4.1 Chlamydia trachomatis

**FIGURE 1:**
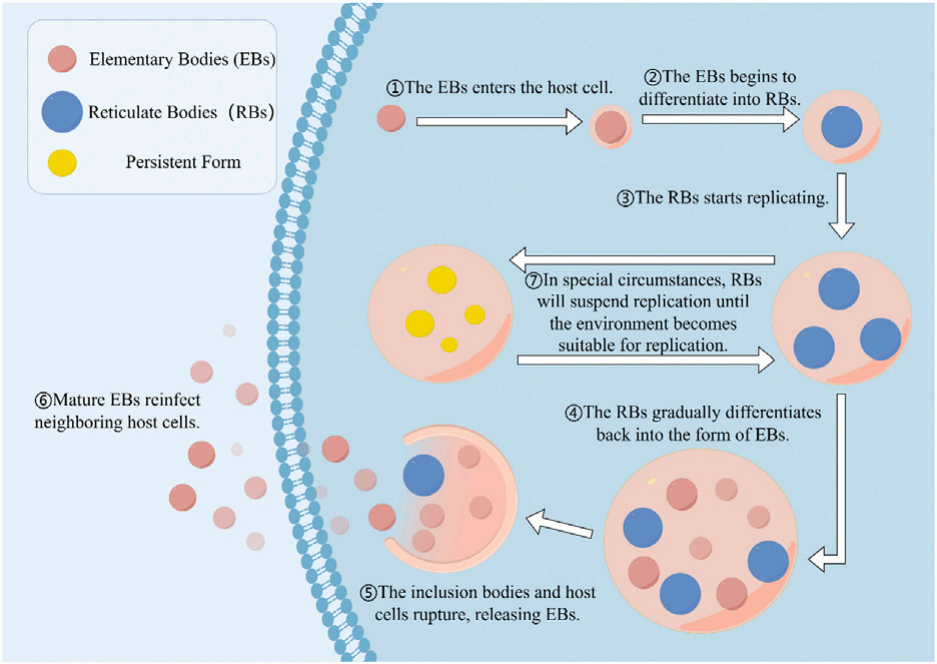
The developmental cycle of *Chlamydia trachomatis*. Infectious EBs (elementary bodies) enter host cells, whereupon the EBS begin to differentiate into replicable RBs (reticulate bodies). During the RBs replication stage, metabolites of the host cells provide the material basis for the pathogen. After several rounds of replication, RBs redifferentiate into EBs and are released upon rupture of the cell membrane or lysis of the host cell, subsequently infecting other cells. In stressful environments, RBs will cease replication and transform into a stable form until the stress disappears.

*C. trachomatis* primarily targets urogenital epithelial cells, specifically the columnar and transitional types, causing extensive functional disruptions and structural damage to the fallopian tubes. Such damage often leads to the formation of pelvic adhesions. The persistence of these infections is greatly influenced by the pathogen’s unique biphasic life cycle. This cycle involves alternating between two distinct morphological states: the Elementary Body (EB) and the Reticulate Body (RB) (Abdelrahman and Belland, 2005). The EB is the extracellular, metabolically inactive form that plays a critical role in the dissemination of the infection and the initial invasion of host cells. It adheres to susceptible host cells and penetrates them effectively. Following entry, the EB is encapsulated by a phagosome from the host cell, which initiates its transformation into the RB. This transformation marks a pivotal shift in the pathogen’s lifecycle as it moves from an inert state into an active intracellular replication phase, characterized by increased bacterial metabolism and the transition from the EB to the RB form (Belland et al., 2003) (Figure 1). The complete developmental cycle, encompassing the conversion from RB back to EB, is completed within approximately 40–48 h, at which point newly formed EBs are released to infect adjacent cells (Lee et al., 2018). While antibiotic therapy is generally effective against *C. trachomatis* infections, the pathogen can re-initiate its developmental cycle following the discontinuation of treatment, resulting in persistent reinfection and an elevated risk of antibiotic resistance (Kintner et al., 2014). Consequently, the development of innovative strategies to prevent recurrent *C. trachomatis* infections represents an urgent and critical area of research.

Research has highlighted the potential of certain metabolites to disrupt the life cycle of *C. trachomatis*. Among these, Andrographolide (Andro), a diterpenoid lactone isolated from the plant *Andrographis paniculata* [Acanthaceae; *A. paniculata* (Burm.f.) Wall. ex Nees], has been shown to effectively inhibit the infection process of *C. trachomatis* within cultured human cervical epithelial cells. Andrographolide’s mechanism is multifunctional in its interaction with the pathogen and the host cells. It notably inhibits the transformation of *C. trachomatis* from its metabolically active RB stage to its infectious EB form. Furthermore, Andrographolide significantly diminishes the release of pro-inflammatory cytokines such as IL-6, C-X-C motif chemokine ligand 8 (CXCL-8), and interferon-gamma- IP-10 by epithelial cells triggered by the infection (Hua et al., 2015). This study have investigated the cytotoxicity of Andro *in vitro*, using various concentrations to identify a range with minimal cytotoxic effects. However, the role of Andro-induced downregulation of IL-6, CXCL-8, and IP-10 in host immunity—particularly within the context of host-pathogen interactions—remains unclear. Therefore, additional *in vivo* studies are warranted to elucidate these mechanisms.

Baicalin, a flavonoid derived from *Scutellaria baicalensis* [Lamiaceae *; S. baicalensis Georgi*], has demonstrated notable inhibitory effects against *C*. *trachomatis* serovar D infection in Hep-2 cells. Huang Hao et al. reported that baicalin significantly reduces *C. trachomatis* infection in a dose-dependent manner, achieving over 75% inhibition at concentrations ranging from 0.12 to 0.48 mg/mL (Hao et al., 2009). Furthermore, baicalin treatment was shown to upregulate RFX5 expression while concurrently downregulating the chlamydial protease-like activity factor (CPAF), a protease implicated in the degradation of RFX5. These findings highlight CPAF as a primary molecular target of baicalin, underscoring its critical role in modulating RFX5 expression. Subsequent investigations further revealed that baicalin effectively suppressed the overexpression of Toll-like receptor 2 (TLR2) and Toll-like receptor 4 (TLR4) in *C. trachomatis*-infected mice cervical tissues (Hao et al., 2011). In addition, baicalin significantly reduced the number and size of chlamydial inclusion bodies, inhibited the activation of CPAF, and prevented the degradation of RFX5 and upstream stimulatory factor 1 (USF-1). Notably, baicalin also mitigated the downregulation of major histocompatibility complex class I (MHC I) and CD1d expression on the surface of infected cells. This suggests that baicalin plays a crucial role in preserving antigen presentation and enhancing the host immune response during *C. trachomatis* infection (Hao et al., 2013). Nevertheless, toxicity assessments of baicalin were not performed in the referenced studies, and further investigation is required to determine its safety profile.

### 4.2 Neisseria gonorrhoeae

*N. gonorrhoeae* is a Gram-negative bacterium and one of the primary pathogens responsible for PID. *N. gonorrhoeae* expresses three major outer membrane proteins: the porin ion channel protein (PorB), opacity-associated (Opa) protein, and reduction-modifiable protein (Rmp). In addition to these, it possesses two key pathogen-associated molecular patterns (PAMPs): lipooligosaccharide (LOS) and type IV pili (Edwards and Apicella, 2004). Allelic variation is common among PorB isoforms across gonococcal strains, predominantly occurring within the surface-exposed loops of the porin molecules (Garvin et al., 2008). In terms of antigenic variation, a single *N. gonorrhoeae* strain can encode up to 11 distinct opa genes, enabling the constitutive expression of multiple Opa protein variants (Bhat et al., 1991). These characteristics allow *N. gonorrhoeae* to establish persistent infections, as the human immune system struggles to develop effective immunological memory against the pathogen. Furthermore, macrophages infected with *N. gonorrhoeae* exhibit diminished capacity to stimulate T-cell responses (Escobar et al., 2018). Notably, during macrophage infection, the expression levels of the antibody receptor CD64 fail to elevate, which is hypothesized to attenuate antibody-dependent cellular cytotoxicity (ADCC) responses against the bacterium (Ortiz et al., 2015). Consequently, exploring botanical agents with antimicrobial activity as sources of antimicrobial metabolites—through mechanisms such as disrupting gonococcal adhesion or activating anti-inflammatory pathways to inhibit infection—holds promise for developing novel therapeutic strategies against *N. gonorrhoeae*.

*Coscinium fenestratum* [Menispermaceae; *C. fenestratum* (Gaertn.) Colebr.], traditionally sourced from regions spanning India to Malesia, has been identified for its medicinal properties, particularly in combating bacterial infections. Prominent *in vitro* experiments have revealed that the stem extract of *C. fenestratum* possesses robust antigonococcal properties, demonstrating a minimum inhibitory concentration (MIC) of 47.39 μg/mL against the *N*. *gonorrhoeae* ATCC 49226 strain. Following these findings, the extract was processed through column chromatography, resulting in the isolation of berberine, a significant metabolite. Subsequent evaluations of berberine confirmed its substantial antigonococcal efficacy, with it exhibiting average MIC values of 13.51 μg/mL against the *N*. *gonorrhoeae* ATCC 49226 and 17.66 μg/mL against various clinical isolates. Additionally, an acute toxicity assessment conducted on the crude methanolic extract from the *C. fenestratum* stem in laboratory mice showed no signs of mortality or adverse symptoms throughout the duration of a 1-week observational period (Chomnawang et al., 2009). The acute toxicity assessment of the methanolic crude extract derived from *C. fenestratum* stems revealed no mortality or abnormal clinical signs in either control or treatment groups throughout the 7-day observation period, indicating a favorable safety profile for this botanical preparation. In this study, although screening of plants with anti-gonococcal activity was performed, and comparative analyses with reference metabolites preliminarily identified the primary active components in the herbal extract, along with acute toxicity tests in animal models, the potential presence of additional antimicrobial metabolites in the extract remains to be elucidated. Furthermore, the antimicrobial mechanisms, as well as the safe dosage and risks associated with long-term use, require further exploration.

*Helichrysum caespititium* [Asteraceae; *H. caespititium* (DC.) Sond.], a botanical drug native to the regions extending from Zimbabwe to South Africa, has garnered attention for its significant pharmacological properties. Earlier research confirmed that its non-polar extract possesses exceptionally strong antimicrobial and antioxidant properties (Mamabolo et al., 2017). Subsequent studies have built upon these findings, underscoring the efficacy of *H. caespititium* in treating gonorrheal infections and exhibiting antimicrobial, anti-quorum sensing, and antibiofilm capabilities. Notably, one metabolite in particular, 10-methyl-8-(propan-17-ylidene)naphthalen-9-yl)-11-vinyl-14-hydroxyfuran-16-one (CF6), isolated from the chloroform extract of the plant, showed potent activity against *N*. *gonorrhoeae*. The MIC of CF6 was determined to be 60 μg/mL against the reference strain *N*. *gonorrhoeae* ATCC 49226. The metabolite’s anti-quorum sensing potential is particularly notable, believed to be due to its ability to competitively bind to the receptor proteins of *Chromobacterium violaceum*, potentially blocking the active binding sites essential for bacterial communication. Furthermore, CF6 effectively inhibited the cell attachment of *N. gonorrhoeae*. The underlying mechanism of this inhibitory effect may involve CF6 disrupting the cohesiveness of bacterial extracellular polysaccharides, thereby reducing bacterial extracellular polysaccharides structural integrity and impairing cell attachment (Bassey et al., 2021). Thus, this botanical drug and its metabolites hold potential for development as therapeutic agents against *N*. *gonorrhoeae*.

*Ocimum tenuiflorum* [Lamiaceae; *O. tenuiflorum* L.], the native range of this species is Tropical and Subtropical Asia to W. Pacific. *Ocimum tenuiflorum* has been traditionally used since ancient times to treat various diseases, including those caused by microorganisms. Bioassay-guided purification of the hexane extract from the leaves of *O. tenuiflorum* led to the isolation of H12c as the active metabolite. Characterization identified H12c as eugenol, which exhibited a MIC range of 85–256 mg/L. Notably, this study revealed a strong inhibitory effect of eugenol against both common and multidrug-resistant strains of *N*. *gonorrhoeae*. Furthermore, the 50% lethal dose (LD50) of H12c was determined to be 2 g/kg body weight in rats, indicating a low toxicity profile (Shokeen et al., 2008). Given its efficacy and low toxicity, eugenol represents a promising candidate for clinical development to address the rising prevalence of resistant *N. gonorrhoeae* isolates.

**FIGURE 2:**
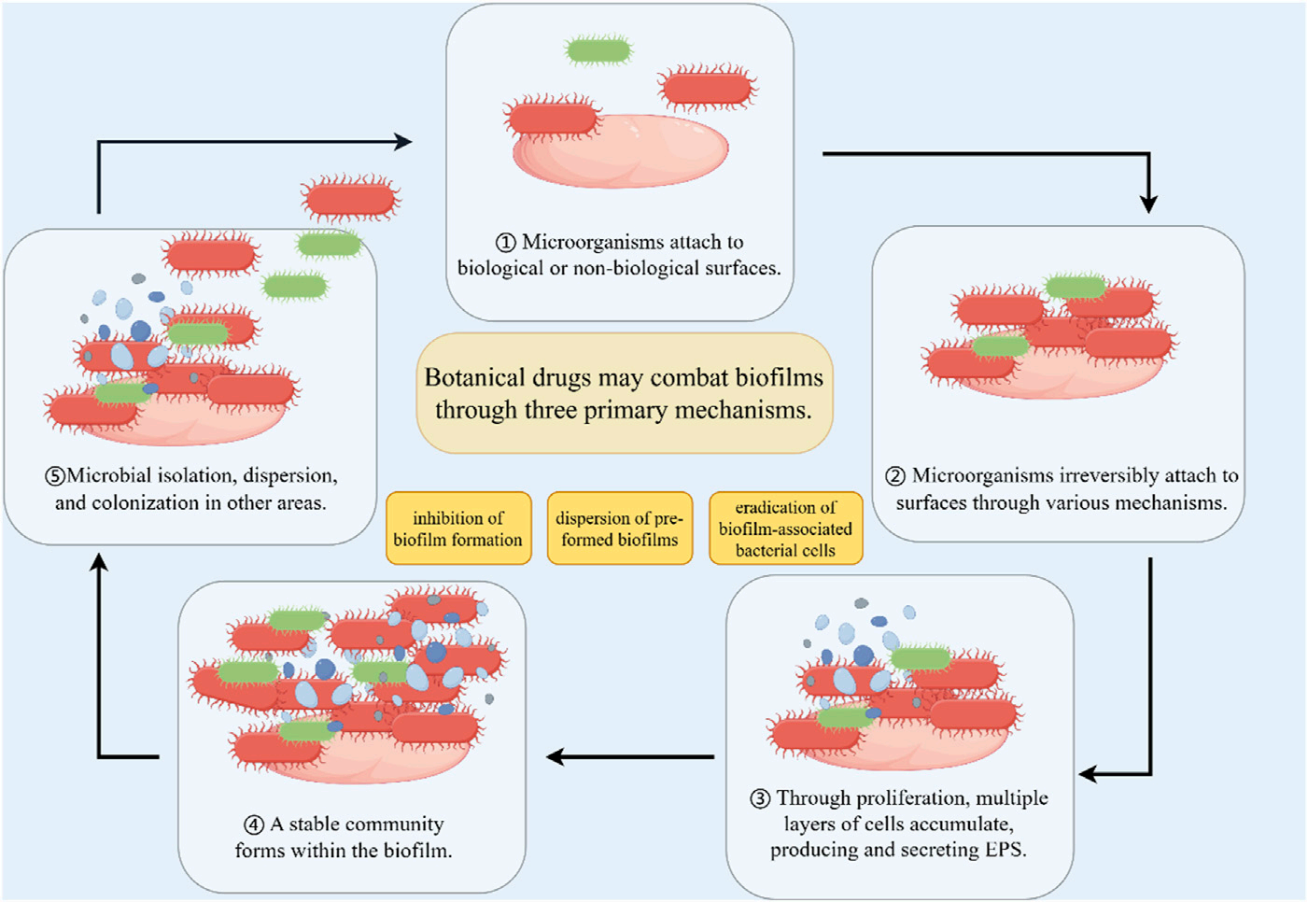
Biofilm and biofilm inhibition. The process of microbial biofilm formation. Microorganisms first reversibly attach to the surface of cells or substances. Subsequently, microbial cells irreversibly colonize through flagella and other mechanisms. A sufficient number of microorganisms form multiple layers of cells and accumulate outside the cells, while producing extracellular polymeric substances (EPS). Form a stable three-dimensional community during the development process. Microorganisms can diffuse from aggregated biofilms and return to a planktonic state.

*Magnolia officinalis* [Magnoliaceae; *M. officinalis* Rehder and E.H.Wilson], a traditional Chinese botanical drug, is derived from the dried bark, root bark, and branch bark of *M. officinalis*, a plant belonging to the Magnoliaceae family. As documented in the *Compendium of Materia Medica* (Bencao Gangmu), it is named “Houpo” due to its thick bark and robust wood structure. Honokiol, a key phenolic metabolite extracted from the bark of *M. officinalis*, exhibits diverse biological activities. Previous studies have demonstrated that honokiol exerts immunomodulatory effects by enhancing interferon production and simultaneously reducing reactive oxygen species (ROS) and pro-inflammatory cytokine levels in macrophages infected with Staphylococcus aureus(Choi et al., 2015). Recent research has provided further insights into its mechanisms of action. Detailed investigations reveal that honokiol reduces the production of hydrogen peroxide (H_2_O_2_) and inhibits the phosphorylation of ERK1/2 in macrophages infected with *N*. *gonorrhoeae*. Furthermore, honokiol suppresses the expression of the pro-inflammatory mediator interleukin-6 (IL-6) and inducible nitric oxide synthase (iNOS) induced by *N. gonorrhoeae* through a pathway independent of NLRP3 inflammasome activation (Hua et al., 2024). On the other hand, this study has certain limitations, primarily the absence of an animal study to assess the efficacy of honokiol against *N. gonorrhoeae* in a living organism. Further research, including *in vivo* studies, is needed to provide a more comprehensive understanding of honokiol’s potential as a therapeutic agent against *N. gonorrhoeae* infections (Figure 2) (Table 2).

**TABLE 2:** Antibacterial effect of Botanical drugs and its Metabolites on exogenous pathogen.

	Botanical drugs	Metabolites/Extracts	Preparation method	Identification method	Controls	Cells/Animals	Model	Dose (concentration)	Duration of administration	Minimum inhibitory concentration (MIC)	Results	References
*Chlamydia trachomatis*	*Andrographis paniculat*a	Andrographolide (Andro)	Andrographolide was purchased from Sigma-Aldric	/	dimethylsulfoxide (DMSO)	*C. trachomatis* strains (*in vitro*)	*/*	10 μl	44 h	/	Perturb *C. trachomatis* transition from the metabolically active reticulate body to the infectious elementary body and concurrently reduce the production of a proinflammatory mediator by epithelial cells *in vitro*.	Hua et al. (2015)
	*Scutellaria baicalensis*	Baicalin	Baicalin (HPLC content > 98.0%) from Chongqing Green Valley Bio-tech Co. LTD	/	/	*C. trachomatis* serovar D (*in vitro*)	*/*	0.12, 0.24, 0.48 mg/mL of Baicalin	24 h	/	Over 75% reduction in infection by serovars D was obtained at concentrations of the Baicalin between 0.12 and 0.48 mg/mL. RFX5 and CPAF were up-regulated and down-regulated respectively by Baicalin.	Hua et al. (2009)
	*Scutellaria baicalensis*	Baicalin	Baicalin is produced by Sichuan Guanghan Weikang Plant Chemical Co., Ltd., with a content of 97.6%	/	Azithromycin group	Standard strain of *Chlamydia trachomatis* type E (*in vitro*); mice model	An animal model of CT infection of the reproductive tract of a mice model	10, 25, 50 mg/kg^-1^ of Baicalin	6 days	/	Baicalin can effectively inhibit the number of *Chlamydia trachomatis* infections and the high expression of TLR2 and TLR4 in the cervical tissue of mice infected with *Chlamydia trachomatis*.	Huang et al. (2011)
	*Scutellaria baicalensis*	Baicalin	Baicalin is produced by Sichuan Guanghan Weikang Plant Chemical Co., Ltd., with a content of 97.6%	/	Negative: dimethylsulfoxide (DMSO)	Standard strain of *Chlamydia trachomatis* type E (*in vitro*)	/	27, 0.54, 1.08 mmol·L^-1^ of Baicalin	18 h	/	Baicalin significantly inhibited the number and size of inclusion bodies formed, reduced the content of activated CPAF, inhibited the degradation of RFX5 and USF-1, and inhibited the downregulation of MHCⅠ and CD1d expression on the surface of infected cells.	Huang et al. (2013)
*Neisseria gonorrhoeae*	*Coscinium fenestratum*	Methanolic crude extract	Solutions of methanolic Coscinium fenestratum crude extract, standard berberine chloride dihydrate and separated pure berberine were prepared in methanol at the concentrations of 200, 44 and 10 μg/μl, respectively. Berberine was separated from the methanolic crude extract of *Coscinium fenestratum *by column chromatography.	Quantitative analysis of berberine content in the crude methanolic extract of *Coscinium fenestratum* was evaluated by a validated TLC-densitometric method	Purified berberine Positive: solution of ceftriazone sodium	*Neisseria gonorrhoeae* ATCC 49226 and 11 clinical isolates (*in vitro*)	/	Methanolic extract of *Coscinium fenestratum* 200 μg/ml,purified berberine 10 μg/ml, 3 μg/ml	24h	Purified berberine (13.51 ± 0.25 μg/ml), *Coscinium fenestratum* (47.39 ± 0.58μg/ml)	This study discovered notable anti-gonococcal activity in *Coscinium fenestratum* among various Thai medicinal plants. Through comparative analysis with purified berberine, the extract was preliminarily identified to contain berberine as one of its main metabolites.	Chomnawang et al. (2009)
				Positive: solution of ceftriazone sodium			3 μg/ml		0.0034 ± 0.0007μg/ml	0.0034 ± 0.0007μg/ml		
	*Helichrysum caespititium*	10-methyl-8-(propan-17-ylidene)naphthalen-9-yl)-11-vinyl-14-hydroxyfuran-16-one (CF6)	Isolation from the Chloroform extract, using column chromatography method	UPLC-QTOF-MS analyses	Negative: 1% DMSO	*Neisseria gonorrhoeae* ATCC 49981 (*in vitro*)	*/*	1 mg/mL	24 h	60 µg/mL	The anti-gonococcal therapeutic effects of non-polar extracts from Helichrysum caespititium were primarily attributed to their anti-biofilm activity, whereas their anti-quorum sensing activity exhibited limited efficacy.	Bassey et al. (2021)
					Positive: ciprofloxacin			1 μg/mL		1 μg/mL		
	*Ocimum tenuiflorum*	Eugenol	Extracted with hexane,and the hexane extract were purified using silica gel G (60–120 μ mesh size) column chromatography.	LC–MS/MS–TOF analyses	Positive: penicillin Positive: ciprofloxacin	*Neisseria gonorrhoeae *clinical isolates and World Health Organization (WHO) strains	*/*	0.5 IU	NR	85–256 mg/L	The multiresistant strains were inhibited to greater extent than the more susceptible strains. High inhibition of clinical isolates 6–19 (which included multiresistant isolates) by eugenol was also observed.	Shokeen et al. (2008)
								1 μg		Penicillin and ciprofloxacin: NR		
	*Magnolia officinalis*	Honokiol	Acquired from Santa Cruz Biotechnology	/	Negative: 0.1% DMSO	mouse J774A.1 macrophage cell with *N. gonorrhoeae,* ATCC 49226 (*in vitro*)	*/*	5-20 µM	24 h	NR	Honokiol exhibits antimicrobial activity against *N. gonorrhoeae in vitro*. It may augment the phagocytosis and bactericidal capabilities of macrophages against *N. gonorrhoeae*.	(Hua et al. 2024)

## 5 Antibacterial effect of botanical drugs and its metabolites on endogenous pathogens

PID is predominantly caused by ascending infections originating in the vagina or cervix, involving both aerobic bacteria (e.g., *S*. *aureus*, *E*. *coli*) and anaerobic microorganisms (Eschenbach et al., 1975). The polymicrobial nature of PID, characterized by co-infections involving *N*. *gonorrhoeae*, *C*. *trachomatis*, and diverse vaginal microbiota, significantly complicates therapeutic interventions due to synergistic pathogen interactions and enhanced antimicrobial tolerance. Furthermore, the escalating prevalence of antimicrobial resistance (AMR), driven by widespread antibiotic misuse, has intensified the urgency to develop complementary therapeutic strategies targeting these recalcitrant infections. Over the past decade, there has been a growing interest in exploring complementary and alternative therapeutic strategies to combat infections caused by these pathogens. Notably, botanical drugs and its metabolites have demonstrated potential in effectively controlling infections caused by endogenous pathogens, including drug-resistant bacteria. This is achieved through mechanisms such as the inhibition of biofilm formation, inhibition of efflux pumps, suppression of quorum sensing (QS), and prevention of plasmid transfer (Table 3).

**TABLE 3:** Antibacterial effect of Botanical drugs and its Metabolites on endogenous pathogens.

	Botanical drugs	Compound/Extracts	Preparation method	Identification method	Controls	Cells/Animals	Model	Dose (concentration)	Duration of administration	Minimum inhibitory concentration (MIC)	Results	References
Inhibits of biofilms	*Persea americana*	SE: ethanol extract obtained by Soxhlet; MaE: ethanol extract obtained by maceration	—	the phytochemical analysis	—	*Pseudomonas aeruginosa*, *Staphylococcus aureus* (*in vitro*)	*—*	64 μg/mL	24h	*P. aeruginosa* ATCC 9027: SE: 87.0 ± 4.4 μg/mL; MaE: 187.4 ± 9.4; *S. aureus* ATCC 6538: SE: 144.2 ± 5.7 μg/mL; MaE: 159.2 ± 7.9 μg/mL	The anti-biofilm and anti-adhesion activities of the ethanol extracts on *P. aeruginosa* might be associated with the modulation of the quorum sensing system by downregulation of the virulence factors such as *mexT* and *lasA* genes	(Molina Bertrán et al., 2022)
	*Persea americana*	Methanol extract of *Persea americana* seed	The *Persea americana* seeds used in the present study were collected from local area of Dschang in the West Region of Cameroon. 800 g of the ground seeds were soaked into 2.5 L of methanol for 48 h under constant shaking. The mixture was filtered using Whatman no 1 and the filtrate was concentrated using rotary evaporator at 65°C and dried at 40°C in an oven to eliminate the remaining solvent	The chemical screening consisted in performing qualitative chemical tests. The determination of the total phenol content was performed by the spectrophotometric method using the Folin-Ciocalteu reagent. The quantification of flavonoid content was determined using the aluminium trichloride method. The tannin content was determined by the Folin-Ciocalteu method	Amoxicillin trihydrate; Cefotaxim; Levofloxacin hemihydrate; Erythromycin	*Pseudomonas aeruginosa*, *Staphylococcus aureus;* rat model (*in vitro*)	*—*	50 μL	24h	*Staphylococcus aureus* 18 (MIC = 64 μg/mL), *Escherichia coli* 64R (MIC = 128 μg/mL)	The antibacterial activity of the extract is due to the bacterial biofilm inhibition and the perturbation of the bacterial membrane through the leakage of intracellular materials, the inhibition of H + -ATPases pumps	(Ekom et al., 2022)
	*Glycyrrhiza glabra* L	Glabridin	Glabridin was purchased from Sigma-Aldrich Co. LLC	—	—	*Escherichia coli* NBRC15034, *Porphyromonas gingivalis* JCM12257, *Pseudomonas aeruginosa* NBRC13275, *Staphylococcus aureus* NBRC13276, and *Streptococcus mutans* NBRC13955 (*in vitro*)	*—*	200 μL	24h	*Streptococcus mutans* NBRC13955 (MIC = 3.125 μg/mL); *Staphylococcus aureus* NBRC13276 (MIC = 6.25 μg/mL); *Porphyromonas gingivalis* JCM12257 (MIC = 1.562 μg/mL)	Glabridin demonstrated the most potent biofilm eradication activities against *S. mutans*, *S. aureus*, and *P. gingivali*s. The MBECs of glabridin were 25, 50, and 25 μg/mL for *S. mutans*, *S. aureus*, and *P. gingivalis* biofilms	(Tsukatani et al., 2022)
	*Glycyrrhiza glabra* L	Glabridin	Glabridin was purchased from Sigma-Aldrich Co. LLC	—	25 antibiotics including Ampicillin, Cefoxitin and Vancomycin	*Staphylococcus aureus* (*in vitro*)	*—*	12.5, 25, and 50 μg/mL	24h	From 3.12 to 25 μg/mL	Glabridin was found to reduce the MIC of different antibiotics such as norfloxacin, oxacillin, and vancomycin by up to 4-fold, while the MIC of glabridin itself was found to be reduced by up to 8-fold in the presence of antibiotics	(Singh et al., 2015)
	*Glycyrrhiza glabra* L	Glabridin	Glabridin were obtained from Aladdin (Shanghai, China)	—	9 antibiotics including Ampicillin, Vancomycin and Levofloxacin	*Escherichia coli strains* (*in vitro*)	*—*	100 μL	24h	>64 μg/mL	Glabridin synergistically suppressed *E. coli* biofilms formation and eradication with colistin	(Yang et al., 2024)
	*Carum copticum*	Zone of inhibition (ZOI) for methanolic and ethanolic extracts of *C. copticum*	The collected plants were air dried and pulverized into fine powder. Fifty Gram of each powder of plants was macerated into 500 mL of ethanol and methanol solvents	GC-MS	—	*Staphylococcus aureus* (*in vitro*)	*—*	12.5–50 mg/mL	24h	*S. aureus* : Methanolic extract (MIC = 25 mg/mL); Ethanolic extract (MIC = 3.125 mg/mL)	*C. copticum* extracts had ability to inhibit of bacteria and inhibit biofilm	(Mohammadi et al., 2019)
	*Centella asiatica*	Madcapsic Acid (MA)	The powder of *C. asiatica* leaves (2 gm) were subjected to ultrasound treatment using ultrasonicator	HPLC analysis	DMSO	*Staphylococcus aureus* (*in vitro*)	*—*	31.25 μg/mL	28h	The MIC values for *Staphylococcus aureus*, Methicillin-resistant *Staphylococcus aureus*, *Escherichia coli*, *Pseudomonas aeruginosa*, *Bacillus subtilis*, and *Bacillus megaterium* were 31.25, 62.5, 250, 125, 62.5, and 62.5 μg/mL	MA could destroy the integrity of the cell membrane and cell wall of *Staphylococcus aureus*	(Wei et al., 2023)
Inhibits outflow pumps	*Zanthoxylum nitidum*	6-acetonyl-dihydrofagaridine	The dried roots of *Z. nitidum* (10 kg) were crushed and extracted with 85% ethanol (30 L, 8 h × 3) at 55°C under reflux conditions. The alkaloid-rich moiety was chromatographed using a silica gel column and gradient of petroleum ether-acetone to afford six fractions (Fr. Ⅰ–Ⅵ)	column chromatography	Berberine; methicillin; ampicillin; vancomycin	*S. aureus (ATCC 25923 AS1.2386), MRSA* (*in vitro*)	*—*	34.8 mg	24h	6-acetonyl-dihydrofagaridine (MIC: 16–32 μg/mL); 6-acetonyl-dihydrofagaridine + ampicillin (MIC: 4–8 μg/mL)	6-acetonyl-dihydrofagaridine showed anti-MRSA bioactivity and synergistic action with ampicillin *in vitro* indicated.The compound inhibited the efflux of the drug by combining with ampicillin	(Zeng et al., 2022)
	red ginseng	ginsenoside 20(S)-Rh2	20(S)-ginsenoside Rh2 (purity>98%) was purchased from Jilin University (Changchun, China)	—	Ciprofloxacin	*Staphylococcus aureus* (*in vitro*)	*S. aureus* infected peritonitis mice	50–100 mg/kg	24h	Ciprofloxacin (CIP) +Rh2 (10 μM) (MIC: 0.25–32 mg/L)	Rh2 significantly promoted the accumulations of CIP in *S. aureus*, and inhibited the *NorA* mediated efflux of pyronin Y	(Zhang et al., 2014)
	Persea lingue	kaempferol-3-O-α-L-(2,4-bis-E-p-coumaroyl)rhamnoside	Dried ground leaves (700 g) were ultrasonicated three times with 96% ethanol 1:10 (mass to volume) for 30 min and the mixture was filtered	vacuum liquid chromatography	reserpine	*Escherichia coli* (*in vitro*)	—	22 mg	NR	25 mg/L	kaempferol-3-O-α-L-(2,4-bis-E-p-coumaroyl)rhamnosideacts through inhibition of the *NorA* efflux pump	(Holler et al., 2012)
	Arrabidaea brachypoda	Brachydins BR-B	A Shimadzu model HPLC system (Shimadzu Corp., Kyoto, Japan) was used, and mass spectrometric experiments were performed	Mass spectrometric experiments were performed on LCQ Fleet Equipment (Thermo Scientific) equipped with a direct sample insertion device via flow injection analysis (FIA). The studied matrix was analyzed by electrospray ionization (ESI), the multi-stage fragmentation (MS2, MS3 and MSn) was performed with an ion interface trap (IT)	Norfloxacin	*S. aureus* And C. albicans (*in vitro*)	—	128 μg/mL	24h	*S. Aureus* (MIC ≧1024 μg/mL) , *C. albicans* (MIC = 141 μg/mL)	BR-B potentiated the activity of Norfloxacin against SA1199-B. BR-B also was able to modulate the resistance to EtBr against SA1199-B. BR-B caused intracellular accumulation of EtBr behavioring as a *NorA* inhibitor	(de Sousa Andrade et al., 2020)
Inhibits quorum sensing	*Polydora serratuloides*	keto-hirsutinolides	Compound 1 was isolated as a brown crystalline solid with a melting point of 95–96 C	—	—	*S. aureus* (*in vitro*)	—	0.33 mg/mL	24h	0.33–5.25 mg/mL^-1^	Keto-hirsutinolides exhibit QSI activity within a concentration range of 0.33–5.25 mg/mL^-1^	(Aliyu et al., 2021)
	*Campsis grandiflora*	The ethanol extract of *Campsis grandiflora* flowers (CFEE)	CFEE was purified by macroporous resin HPD600, and the polyphenol content in the extract was determined. Total polyphenol content of the crude ethanol extract from *C. grandiflora* was 247.66 mg GA/g, and that of the CFEE prepared in this study was 480.4 mg GA/g. After purification by macroporous resin, the polyphenols content of CFEE was nearly doubled	UPLC-MS/MS	—	*Escherichia coli* K-12 (*in vitro*)	*—*	50, 100, and 200 μg/mL CFEE	48h	1000 μg/mL	CFEE has been shown to inhibit the swarming motility of *Escherichia coli* K-12, modulate biofilm structure through the QS system, and exhibit biofilm-clearing effects on mature biofilms	Zhang et al. (2020)
Inhibits of Plasmid transfer	*Mallotus philippensis*	Rottlerin [5,7-dihydroxy-2,2-dimethyl-6-(2,4,6-trihydroxy-3-methyl-5-acetylbenzyl)-8-cinnamoyl-1,2-chromene] and a red compound [8-cinnamoyl-5,7-dihydroxy-2,2,6-trimethylchromene]	Red powder (500 g) was exhaustively extracted by cold agitation with solvents of increasing order of polarity (2 L of hexane, chloroform and methanol). The solutions were placed in an ultrasonic bath for 48 h. The resulting extracts were dried under vacuum on a rotary evaporator and were stored in a refrigerator for further analysis	Chromatographic separations using thin layer chromatography (TLC), column chromatography and vacuum liquid chromatography (VLC)	—	*S. aureus* And *Escherichia coli* (*in vitro*)	—	100 mg/L	24h	Rottlerin (MIC: 1–512 mg/L); red compound (MIC: 32–256 mg/L)	Both compounds strongly inhibited transfer of the plasmids pKM101, TP114, pUB307 and R6K	(Oyedemi et al., 2016)

### 5.1 Inhibits efflux pumps

**FIGURE 3:**
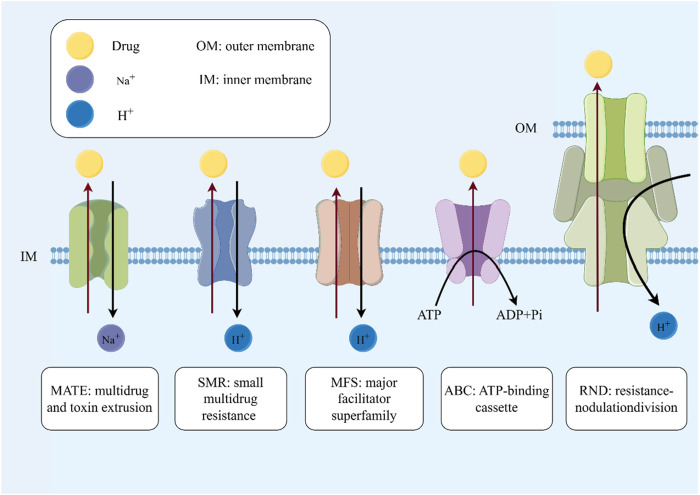
Inhibits outflow pumps. The mechanism of efflux pumps: the five efflux pumps shown in the figure are important mechanisms for bacteria to develop drug resistance. They can transport substrates while expelling drugs or other substances that are not conducive to bacterial growth, thereby increasing drug resistance. It is worth noting that the ABC pump is an efflux pump system that relies on ATP hydrolysis. The energy provided by ATP hydrolysis is used to eliminate various substances from the cell, including antibiotics, metabolic waste, etc.

Efflux pumps in the cytoplasmic membrane are vital transport proteins that expel various substrates from the cytoplasm, as described by Hersch and colleagues (Hersch et al., 2020). These pumps are crucial for ejecting antimicrobial agents from bacterial cells, lowering their intracellular levels and promoting antibiotic resistance development. In *S*. *aureus*, the multidrug efflux pump proteins *QacA*, *NorA* and *Smr* are well-known (Foster, 2016; Jang, 2016). Research indicates the operation of multiple active efflux systems in MRSA, including *QacB*, *QacJ* and *Smr*, which significantly contribute to its multidrug resistance (Xiao Neng et al., 2007) (Figure 3).

*Zanthoxylum nitidum* [Rutaceae; *Z. nitidum* (Roxb.) DC.], belonging to the family Rutaceae and known for its traditional medicinal uses in China for detoxification, pain relief, and blood stasis, contains 6-acetonyl-dihydrofagaridine. This novel coumarin, extracted from the plant’s roots, has shown effectiveness in reducing the MIC by impeding drug efflux in combination with ampicillin (Zeng et al., 2022). However, this study mainly focused on evaluating their anti-MRSA activity, while lacking an in-depth, systematic exploration of specific antibacterial mechanisms, such as effects on bacterial cell membranes, cell walls, or the regulation of related gene expression.

Another notable, 20(S)-Rh2 from *Panax ginseng* [Araliaceae; *P. ginseng* C.A.Mey. ], has exhibited potential in augmenting antibiotic effects by targeting the *NorA* efflux pump in *S*. *aureus*, enhancing the intracellular presence of ciprofloxacin (CIP) and thus its antibacterial properties (Zhang et al., 2014). Although studies have hypothesized 20(S)-Rh2 as a potential inhibitor of *NorA* pumps, there may be a lack of direct molecular-level evidence, such as specific structural binding data, protein expression levels, or in-depth mechanistic studies such as gene regulation.

From the Chilean Huilliche medicinal repertoire, *Persea lingue* [Lauraceae; *P. lingue* (Ruiz and Pav.) Nees], also from the Lauraceae family, has been studied for its content of kaempferol-3-O-α-L-(2,4-bis-E-p-coumaroyl)rhamnoside. In enriched everted membrane vesicles of *E*. *coli*, this metabolite inhibits the *NorA* efflux pump, significantly boosting the effectiveness of ciprofloxacin against strains overexpressing *NorA* (Holler et al., 2012). Although this study has identified inhibitory activity against *NorA* pumps, the specific molecular mechanism of action has not been explored in depth.

From the Brazilian Cerrado, *Fridericia platyphylla* [Bignoniaceae; *F. platyphylla* (Cham.) L.G.Lohmann] yields chalcones that impact bacterial resistance by binding to *NorA* and *MepA* efflux pumps at sites also targeted by norfloxacin. This binding inhibits the efflux activity, suggesting a promising role for these metabolites in combination therapies aimed at *NorA* and *MepA*-mediated resistance in *S. aureus* (de Sousa Andrade et al., 2020). However, this study did not provide data on the pharmacokinetics (e.g., absorption, distribution, metabolism, excretion) and safety (toxicology) of extracts and isolated botanical metabolites.

### 5.2 Inhibits quorum sensing

**FIGURE 4:**
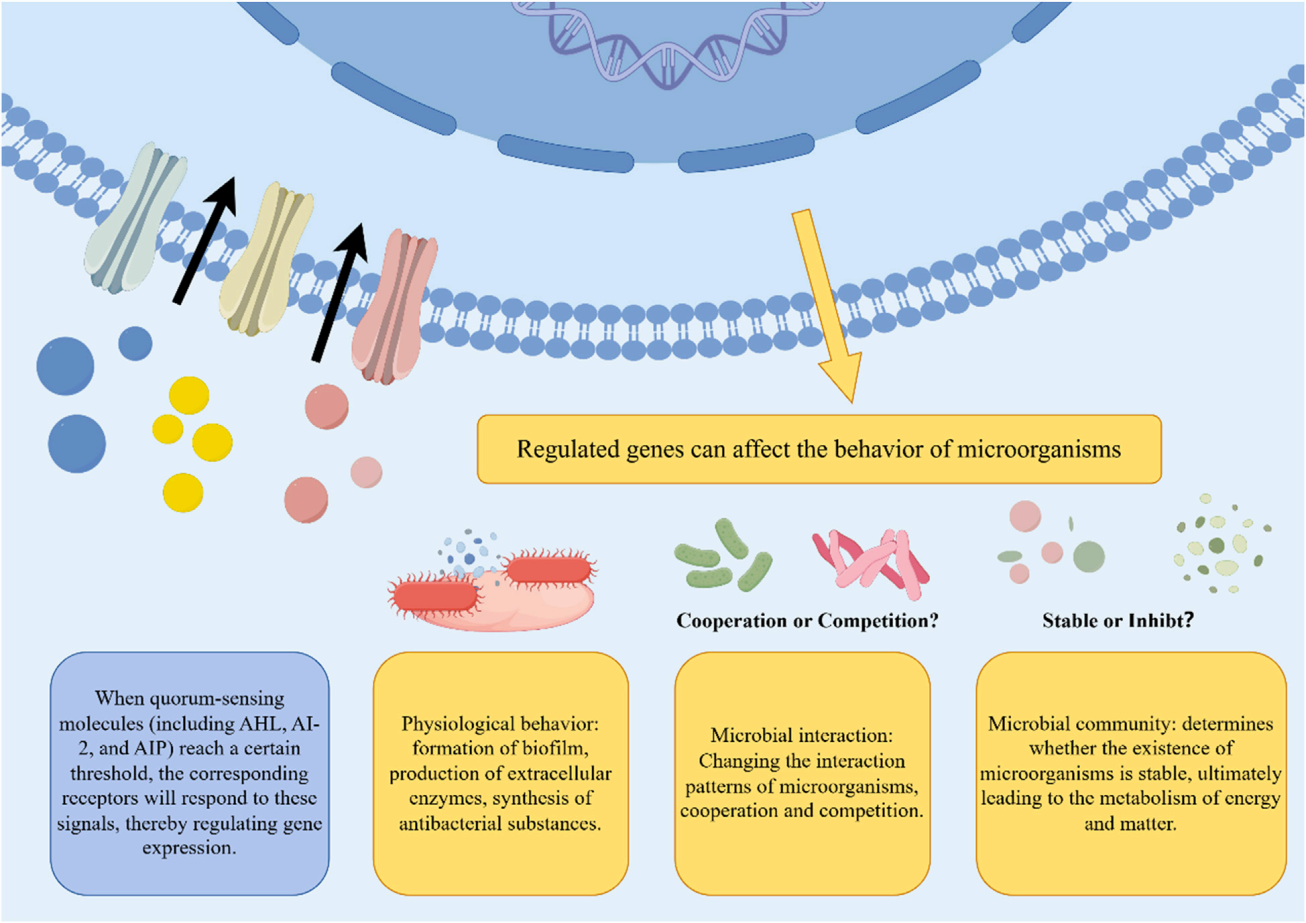
The impact of quorum sensing on microorganisms. Some quorum sensing molecules exist in the extracellular environment. After reaching a certain threshold, the relevant receptors will react to it by regulating genes, ultimately affecting physiological behaviors such as biofilm formation, extracellular enzyme production, and synthesis of antibacterial substances. This will cause the microbial community to change the current balance, and competition or cooperation is uncertain.

Quorum sensing (QS) is a crucial bacterial communication mechanism that regulates collective behaviors such as virulence factor secretion and biofilm formation. This process includes the synthesis, release, and detection of specific signaling molecules on a population-wide scale (Kim et al., 2017). Quorum sensing inhibitors (QSIs), which can reduce bacterial pathogenicity and enhance antibiotic effectiveness while minimizing resistance risks, have been studied extensively (Saeki et al., 2020; Naga et al., 2023) (Figure 4). Natural metabolites from botanical drugs are increasingly recognized as potent sources for QSI development.

In Northern Nigeria, the annual botanical drug *Polydora serratuloides* [*P. serratuloides* (DC.) H. Rob.] is traditionally utilized. Isolation of keto-hirsutinolides from its leaves has demonstrated their activity as QSIs within a concentration range of 0.33–5.25 mg/mL^-1^, indicating their potential in novel antibiotic development (Aliyu et al., 2021). Ongoing studies are needed to explore how these metabolites affect bacterial biofilm formation and the specific pathways through which they modulate the QS system.

The traditional Chinese botanical drug *Campsis grandiflora* [Bignoniaceae; *C. grandiflora* (Thunb.) K. Schum.], also known as “Lingxiaohua”, is noted for its properties in clearing heat and cooling blood as recorded in the *Tang Materia Medica* (*Tang Bencao*). Recent research has shown that the ethanol extract of *C. grandiflora* flowers (CFEE) inhibits violacein production in *C. violaceum* 026 and reduces swarming in *E*. *coli* K-12 and *Pseudomonas aeruginosa* PAO1 in a dose-dependent fashion. Malic acid and succinic acid were identified as effective AI-1 QSIs in these studies, playing key roles in the anti-quorum-sensing activities of CFEE (Zhang et al., 2020). Future investigations should focus on the molecular interactions and specific targets of malic and succinic acids in quorum sensing inhibition.

### 5.3 Inhibits of plasmid transfer

**FIGURE 5:**
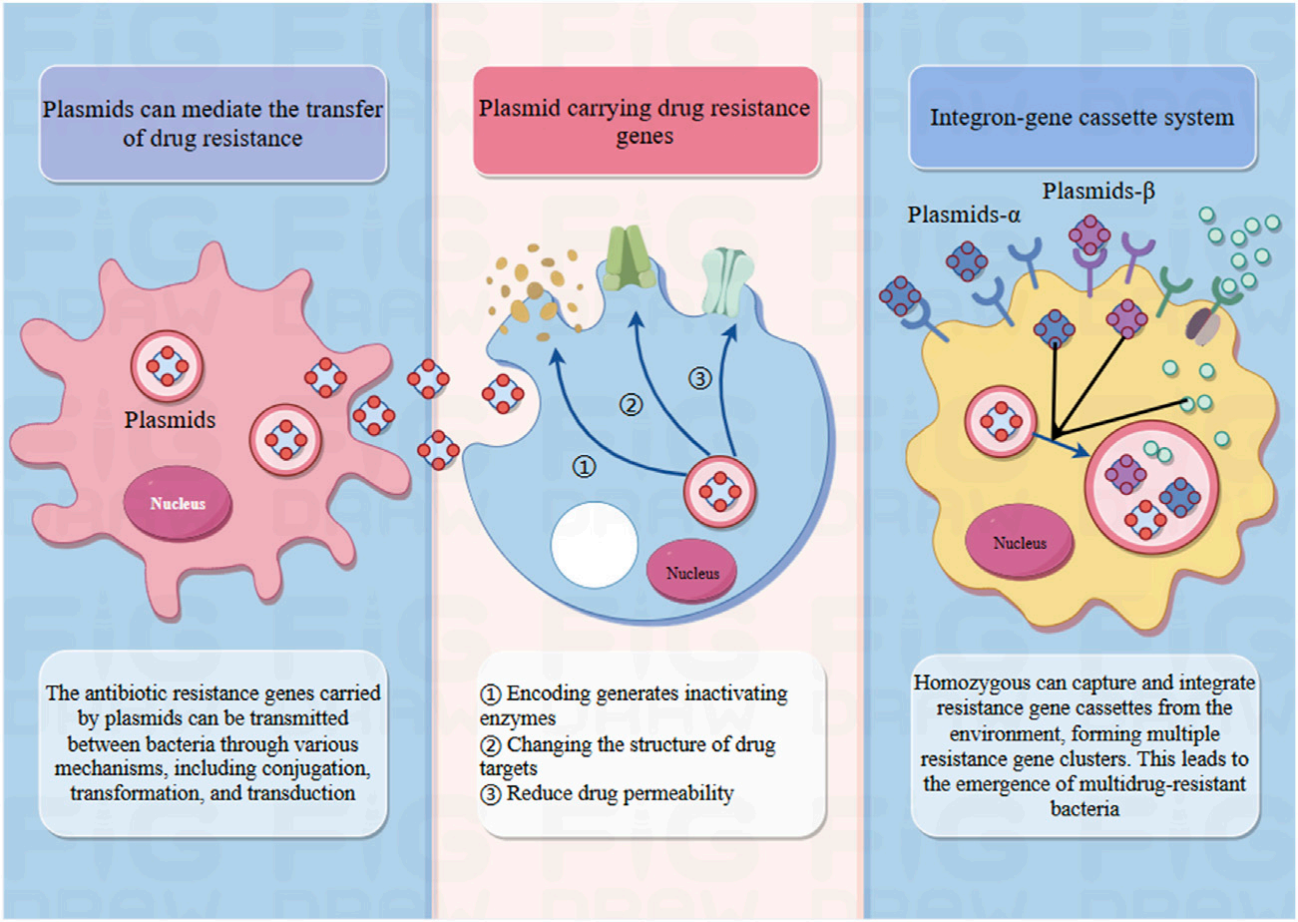
The role of plasmids in drug resistance. Plasmids are scattered DNA fragments in pathogens that do not belong to the nucleus and can perform coding work. Some plasmids carry resistance genes that can encode the production of inactivating enzymes, alter the structure of drug targets, and reduce drug permeability. And the antibiotic resistance genes carried by these plasmids can spread between bacteria through various mechanisms, including conjugation, transformation, and transduction, leading to the transmission of drug resistance. In addition, the integrin gene box system can also be used. Homozygous individuals can capture and integrate resistance gene cassettes from the environment, forming multiple clusters of resistance genes. This has led to the emergence of multidrug-resistant bacteria.

Plasmids play a pivotal role in the dissemination of antibiotic resistance and serve as a key mechanism by which pathogenic strains acquire resistance to antibiotics (de Toro et al., 2014). The activity and horizontal transmission of resistance plasmids markedly increase the prevalence of resistance determinants within bacterial populations, thereby accelerating the emergence and spread of multidrug-resistant bacteria (Mc Carlie et al., 2020). Plasmids play a pivotal role in the dissemination of antibiotic resistance and serve as a key mechanism by which pathogenic strains acquire resistance to antibiotics (de Toro et al., 2014). The activity and horizontal transmission of resistance plasmids markedly increase the prevalence of resistance determinants within bacterial populations, thereby accelerating the emergence and spread of multidrug-resistant bacteria (Mc Carlie et al., 2020) (Figure 5).

*Mallotus philippensis* [Euphorbiaceae; *M. philippensis* (Lam.) Müll.Arg.]*,* a medicinal plant originating from Asia and Australia, is renowned for its diverse therapeutic applications. Research has revealed that the chloroform extract from the leaves of *M. philippensis* contains potent bioactive metabolites, specifically rottlerin [5,7-dihydroxy-2,2-dimethyl-6-(2,4,6-trihydroxy-3-methyl-5-acetylbenzyl)-8-cinnamoyl-1,2-chromene] and a notable red metabolite [8-cinnamoyl-5,7-dihydroxy-2,2,6-trimethylchromene]. These metabolites have been validated for their significant antibacterial activities, particularly effective against a spectrum of clinically important Gram-positive bacteria, including MRSA. Research further demonstrates that at a concentration of 100 mg/L, both rottlerin and the red metabolite ssuccessfully inhibited the conjugal transfer of resistance plasmids such as pKM101, TP114, pUB307, and R6K among strains of *E*. *coli* (Oyedemi et al., 2016). This indicates the potential of these extracts not only as antibacterial agents but also as inhibitors of horizontal gene transfer, which is crucial for preventing the spread of antibiotic resistance. The study underscored the value of these extracts in significantly reducing the dissemination of resistance genes, thereby offering a novel approach to enhance the efficacy of existing antibiotics and mitigate the challenges posed by resistant bacterial strains.

## 6 Synergy with antibiotics

The synergistic effects of combining botanicals with antibiotics have been demonstrated in numerous studies (An et al., 2011; Zuo et al., 2014), particularly in the context of traditional Chinese Medicine (TCM) (Zuo et al., 2012). TCM has been practiced for thousands of years and has proven effective in treating a wide range of infectious diseases. When used in combination with antibiotics, botanicals have been shown to enhance therapeutic efficacy and reduce the development of resistance against pathogens associated with PID (Table 4).

**TABLE 4:** Synergy between botanical drugs or its metabolites and antibiotics.

Botanical drugs	Compound/Extracts	Preparation method	Identification method	Viruses	Controls	Dose (concentration)	Duration of administration	Diameter of the inhibition zone (DIZ)	Minimum inhibitory concentration (MIC)	Minimum bactericidal concentration (MBC)	Antibiotics	Combinatory effects with antibiotics (FIC)	References
*Indigofera suffruticosa*	ether extracts	the leaf powder was homogenized firstly with 200 mL of diethyl ether for 2 h in a mechanical stirrer, kept refrigerated overnight (4°C) and filtered with Whatman no.1 paper. The solvent was then removed under reduced pressure in a rotary evaporator at 45°C	Compounds classes were visualized as aid thin layer chromatography (TLC) on silicagel 60 F254 (Merck), mobile phase standard and Dragendorff, NEU-PEG, KOH-Ethanol, Liebermann-Burchard and vanillin-sulfuric acid reagents, respectively	*Staphylococcus aureus* and some isolated strains of *S. aureus* originally obtained from vaginal secretion, catheter tip, urine sample, blood sample, prostate secretion, wound secretion and ocular secretion (including MRSA strains)	Negative:1% dimethylsulfoxide (DMSO)	100 mg/mL	18h	30.08 ± 2.69 mm	5.9 ± 1.0 mg/mL	16.67 ± 6.2 mg/mL	Erythromycin	0.81 ± 0.18	Bezerra dos Santos et al. (2015)
	Chloroform extracts	The plant material which was not extracted by diethyl ether was then homogenized with 200 mL chloroform						28.79 ± 3.35 mm	4.85 ± 1.6 mg/mL	15.27 ± 7.7 mg/mL		0.68 ± 0.46	
	Acetone extracts	all extraction process was repeated generating the chloroform extract						28.7 ± 3.42 mm	2.16 ± 0.9 mg/mL	7.63 ± 3.8 mg/mL		0.644 ± 0.44	
*Chimonanthus salicifolius*	Ethanol extract	The powder of *C. salicifolius* S. Y. Hu leaves were extracted twice at 40°C followed our previous research: ultrasonic treatment time was 30 min, ultrasonic power was 57.59 W, ethanol concentration was 52.03%, and solid-liquid ratio was 1:23.85. After filtering, the filtrates were pooled, concentrated and further purified by polyamide resin. The adsorbed resin was eluted with gradient aqueous ethanols. The process leaded to in five fractions remained including, 95% ethanol eluate (95% EE), 70% EE and 50% EE, 30% EE, water eluate (WE) at last	Comparing the retention time of reference standards with the HPLC chromatography peaks of the EECS was obtained by DAD spectra	*Staphylococcus aureus*	Negative:DMSO	0.35–45 μg/μL	18h	—	0.35–11.25 μg/μL	5.63–45 μg/μL	Streptomycin	0.13–0.31	Wang et al. (2018a)
				*Escherichia coli CVCC1490*				—	0.70–11.25 μg/μL	5.63–45 μg/μL		0.13–0.63	
*Polyalthia longifolia*	methanol extracts of leaves	The aerial parts of the plants (leaves and stem) were chopped and dried at room temperature in a place protected from direct sunlight. Dried organs were ground, and 30 g of each resulting powder was macerated in 0.5 L of solvent (water, methanol or ethanol) for 72 h at room temperature. The resulting mixture was filtered through filter paper (Whatman No. 3), and the solvent was completely evaporated	UHPLC analysis	*Staphylococcus aureus* ATCC 25923	Negative:DMSO	0.0097–20 mg/mL	12h	—	0.039 mg/mL	0.078 mg/mL	—	—	Savu et al. (2022)
				*Escherichia coli* ATCC 25922				—	10 mg/mL	20 mg/mL			
	Ethanol extracts of leaves			*Staphylococcus aureus* ATCC 25923				—	0.078 mg/mL	0.156 mg/mL			
				*Escherichia coli* ATCC 25922				—	10 mg/mL	20 mg/mL			
	water extracts of leaves			*Staphylococcus aureus* ATCC 25923				—	5 mg/mL	10 mg/mL			
				*Escherichia coli* ATCC 25922				—	5 mg/mL	10 mg/mL			
	methanol extracts of stem			*Staphylococcus aureus* ATCC 25923				—	0.31 mg/mL	20 mg/mL			
				*Escherichia coli* ATCC 25922				—	10 mg/mL	20 mg/mL			
	Ethanol extracts of stem			*Staphylococcus aureus* ATCC 25923				—	0.152 mg/mL	20 mg/mL/			
				*Escherichia coli* ATCC 25922				—	10 mg/mL	20 mg/mL			
	water extracts of stem			*Staphylococcus aureus* ATCC 25923				—	5 mg/mL	20 mg/mL			
				*Escherichia coli* ATCC 25922				—	5 mg/mL	20 mg/mL			
	methanol extracts of leaves			MRSA strain prx-MRSA-2018		0.0003–0.156 mg/mL	24h	—	—	—	penicillin	0.18–2	
	Ethanol extracts of leaves					0.001–0.312 mg/mL						0.18–2	

*Indigofera suffruticosa* [Fabaceae; *I. suffruticosa* Mill.], indigenous to the Antilles and Central America and widely known as “anileira” or “anil,” is traditionally utilized both for indigo dye extraction and various medicinal purposes (Barros and Teixeira, 2008). Its diverse pharmacological benefits, including anti-inflammatory (Chen et al., 2013), anti-convulsant (Almeida et al., 2013) and wound-healing properties (Luiz-Ferreira et al., 2011), have been substantiated through scientific research. Further investigations have demonstrated the antimicrobial prowess of *I. suffruticosa*, particularly via its organic extracts. A study utilizing ether, chloroform, and acetone as solvents revealed significant antimicrobial activities against various strains of *S*. *aureus*, including MRSA (Bezerra dos Santos et al., 2015). Notably, all organic extracts displayed inhibition zones exceeding 30.0 mm against MRSA strains, with diethyl ether extract achieving the highest inhibition at 30.08 ± 2.69 mm. Despite this, solvent-specific differences in effectiveness were not statistically significant (*p* > 0.05). MIC and most potent bactericidal concentration (MBC) for these extracts varied, with ranges from 0.78 to 6.25 mg/mL and 3.12–25.0 mg/mL, respectively. Among these, the acetone extract demonstrated the lowest MIC and MBC values, outperforming ether and chloroform extracts. Moreover, the acetone extract exhibited both bacteriostatic and bactericidal effects, while the others were primarily bactericidal, possibly due to a higher flavonoid content in the acetone extract. When tested in combination with erythromycin, the acetone extract showed varying interactions, ranging from synergistic to non-interactive based on the ratio of drug to extract. Similarly, the chloroform extract displayed synergistic and additive effects in most combinations, with one showing non-interaction. Ether extract combinations mainly showed additive effects. A significant correlation was observed in the interactions of erythromycin with acetone and chloroform extracts. The organic extracts of *I. suffruticosa* represent promising natural candidates for novel anti *S. aureus* formulations, owing to their antimicrobial activity against MRSA strains and the synergistic potential observed when combined with erythromycin. However, the underlying mechanisms of action require further investigation. Additionally, this synergistic effect necessitates validation through *in vivo* studies, while the safe dosage and therapeutic efficacy of these extracts for PID treatment must be confirmed via well-designed clinical trials.

The genus *Chimonanthus* includes rare and endangered plants native to Anhui Province, often found growing in roadside or sparse plateau forests at elevations of 150–400 m. Classified as a dual-use resource for medicine and food (Cheng et al., 2007), *Chimonanthus salicifolius* [Calycanthaceae; *C. salicifolius* S.Y.Hu] are noted in the *Xinhua Compendium of Materia Medica* for their uses in aromatization, clearing heat, detoxification, and preventing colds and influenza (Medicine, 1975). Pharmacological studies have highlighted the plant’s anti-inflammatory, anti-hyperglycemic, anti-cancer and anti-microbial properties (Chen et al., 2017; Sun et al., 2017). While research on this genus remains limited, reports confirm its notable antimicrobial activity (Wu et al., 2017). A preliminary investigation into the interaction between the ethanolic extract of *C. salicifolius* and antibiotics demonstrated promising antimicrobial effects. Specifically, the leaf extract exhibited resistance against foodborne pathogens (Wang et al., 2018a). Further studies explored the synergistic effects of the *C. salicifolius* ethanolic extract combined with streptomycin (Wang et al., 2020). The mixture of ethanol extract and streptomycin effectively inhibited *S*. *aureus* and *E*. *coli*. Mechanistically, the SE extract enhances antimicrobial action by disrupting cell membranes, leading to efflux of nucleic acids, phosphorus, and cytoplasmic contents, while also interfering with total protein synthesis. These findings suggest the potential of *C. salicifolius* as a complementary therapeutic agent in antimicrobial treatments. It seemed that the plant could be developed as antibiotic regulators and the product preventing drug-resistant bacteria. The antimicrobial signaling pathways, toxicological data and require to be further explored.

*Monoon longifolium* [Annonaceae; *M. longifolium* (Sonn.) B. Xue and R.M.K.Saunders] is utilized in traditional medicinal practices across regions from Zimbabwe to South Africa, specifically for treating fever, skin conditions, diabetes, hypertension, and helminthiasis (Wang et al., 2018b). Recent systematic reviews have confirmed the potent antimicrobial effects of both aqueous and methanolic extracts from the leaves of *M. longifolium* (Chanda and Nair, 2010; Faizi et al., 2008). The extracts have pronounced antibacterial efficacy, particularly against *S*. *aureus*, where MICs have reached as low as 0.039 mg/mL. They also exhibit moderate effectiveness against *E*. *coli*, with MIC values observed at 5 and 10 mg/mL (Savu et al., 2022). Additionally, the MBCs for the extracts against *S. aureus* were recorded at 0.078 mg/mL and 0.156 mg/mL for PlLm and PlLe extracts, respectively. These extracts have also been tested against a clinical MRSA isolate, demonstrating strong antibacterial activity with MIC values of 0.312 and 0.624 mg/mL, and an MBC of 10 mg/mL. Regarding antifungal capabilities, the lowest minimum inhibitory or fungicidal concentration (MIC/MFC) noted was 5 mg/mL for various extracts of *M. longifolium*. Moreover, when these extracts were combined with penicillin, there was a notable enhancement in antibacterial activity, reducing MIC values by 33–66 times. *Monoon longifolium* leaf extracts may serve as a promising source of metabolites for developing novel antimicrobial agents to combat antibiotic resistance. However, comprehensive evaluations—including toxicological profiling, preclinical safety assessments, and clinical trials—are imperative to validate their therapeutic potential and ensure translational applicability.

## 7 Limitations

While this review underscores the antimicrobial potential of botanical drugs and their bioactive metabolites against pathogens such as *N. gonorrhoeae* and *C. trachomatis*—positioning them as promising candidates for PID therapy—the majority of cited evidence derives from *in vitro* assays or preclinical animal models. Notably, some studies fail to elucidate critical pharmacological parameters, including safety profiles, effective dosage ranges, or potential adverse effects associated with long-term administration. Furthermore, current clinical applications of these botanicals often rely on anecdotal empirical practices rather than robust clinical trial data, necessitating rigorous validation through standardized interventional studies to establish optimized therapeutic regimens. Additionally, although antibacterial mechanisms such as efflux pump inhibition and membrane disruption are discussed, the molecular pathways underpinning these actions remain poorly characterized. A significant limitation arises from the predominant focus on single bacterial strains (e.g., *S. aureus* isolates or limited drug-resistant variants), with minimal exploration of clinically relevant, multidrug-resistant pathogens. Consequently, the generalizability and translational relevance of these findings to complex human infections remain constrained.

## 8 Future prospects

Botanical drugs, as a vast biochemical reservoir, present tremendous potential for further exploration and development. Future research should prioritize clinical trials to rigorously evaluate the efficacy and safety of botanical drugs and their metabolites in the treatment of PID. Specifically focusing on improving inflammatory markers, relieving patients’ pain, increasing the negative conversion rate of exogenous pathogens associated with PID, alleviating chronic pelvic pain, and evaluating safety indicators. In addition, investigations into the synergistic mechanisms between botanical drugs and antibiotics are necessary to optimize combination ratios. Toxicity assessments and pharmacokinetic studies for such combination therapies should also be undertaken. Furthermore, developing innovative drug formulations is a critical avenue for future research. The integration of nanotechnology or other advanced drug delivery systems could significantly improve the bioavailability and target specificity of botanical drugs, thereby facilitating their translation into clinical practice.

## 9 In conclusion

Botanical drugs and their metabolites have been used to treat infectious diseases since ancient times, serving as a rich reservoir of chemical diversity and antimicrobial activity that continues to influence modern medicine. Over the past decades, numerous studies have highlighted the antimicrobial properties of botanical drugs and their metabolites, partially elucidating their underlying mechanisms, including biofilm inhibition, quorum sensing disruption, efflux pump suppression, and plasmid transfer blockade. These findings underscore the considerable promise of botanical drugs and their metabolites as therapeutic options for PID, offering novel possibilities for antimicrobial treatments.

Despite extensive documentation of the antimicrobial properties of botanical drugs through *in vitro* and *in vivo* studies, clinical research in this area remains limited, constraining their translational potential for practical application. One significant barrier is the insufficient efficacy concentration of plant-derived metabolites. Many metabolites used in TCM contain bioactive substances at relatively low concentrations, which may not be effective in clinical settings. This limitation is particularly pronounced when crude plant extracts are used, where the concentration of metabolites often falls below the therapeutic threshold required for optimal antimicrobial activity.

Additionally, the development of innovative drug delivery technologies, such as nanoparticles, liposomes, or plant-based exosomes, could offer potential solutions to these challenges. Plant exosomes, for example, have emerged as a promising delivery system for plant-derived metabolites. These exosomes can encapsulate bioactive molecules and facilitate their transport across biological barriers, thereby improving the stability and bioavailability of the metabolites. The use of plant exosomes could enable more targeted and sustained release of metabolites, thereby enhancing their therapeutic efficacy and reducing side effects.

In conclusion, botanical drugs and their metabolites represent innovative therapeutic strategies for treating PID while also contributing to efforts addressing the global challenge of antibiotic resistance. Although challenges remain, their remarkable potential underscores the need for continued exploration and development.
