# Supplementary material for: Therapeutic potential of botanical drugs and their metabolites in the treatment of pelvic inflammatory disease
Source: Front Pharmacol. 2025 Apr 10;16:1545917. doi: 10.3389/fphar.2025.1545917 (PMC12018882; doi:10.3389/fphar.2025.1545917)
Supplement: Supplementary file 1 [file Table1.docx]

Supplementary Material

**Supplementary Table1 Literature search method .**

| Pubmed | #1 (Chlamydia trachomatis[MeSH Terms]) OR (Neisseria gonorrhoeae[MeSH Terms])  #2 (Pelvic Inflammatory Disease[MeSH Terms]) OR (Endometritis[MeSH Terms]) OR (Salpingitis[MeSH Terms])  #3 (((botanical drug[Title/Abstract]) OR (herb[Title/Abstract])) OR (herbal medicine[Title/Abstract]) ) OR (pharmacological action[Title/Abstract])  #1 AND #2 AND #3 |
| --- | --- |
| Embase | #1 'Chlamydia trachomatis'/exp/mj OR 'Neisseria gonorrhoeae'/exp/mj  #2 'Pelvic Inflammatory Disease'/exp/mj OR 'Endometritis'/exp/mj OR 'Salpingitis'/exp/mj  #3 'botanical drug' OR 'herb' OR 'herbal medicine' OR 'pharmacological action'  #1 AND #2 AND #3 |
| CNKI | SU='Chlamydia trachomatis'*'Neisseria gonorrhoeae'*'Pelvic Inflammatory Disease'* 'Endometritis'*'Salpingitis' AND TKA='botanical drug'*'herb'*'herbal medicine'*'pharmacological action' |
